# Carob-enriched gluten-free pasta: a sustainable approach for enhancing nutritional, functional, and sensory properties

**DOI:** 10.3389/fnut.2026.1920486

**Published:** 2026-08-12

**Authors:** Héctor Gómez-Llorente, Noelia Castillejo, Joel Girón-Hernández, Piergiorgio Gentile, Isabel Fernández-Segovia, Ana Albors, José Manuel Barat, Édgar Pérez-Esteve

**Affiliations:** 1 Instituto Universitario de Ingeniería de Alimentos – FoodUPV, Universitat Politècnica de València, Camino de Vera s/n, Valencia, Spain; 2 Department of Applied Sciences, Faculty of Health and Life Sciences, Northumbria University, Newcastle upon Tyne, United Kingdom; 3 Centre for Biomaterials and Tissue Engineering, Universitat Politècnica de València, Camino de Vera s/n, València, Spain

**Keywords:** anti-inflammatory activity, antioxidant capacity, celiac diet, *Ceratonia siliqua*, glycemic modulation, oral behavior, plant-based food

## Abstract

**Introduction:**

The development of nutritionally improved gluten-free pasta remains a major challenge due to limitations in texture, functionality, and consumer acceptance. This study aimed to develop a novel carob-enriched gluten-free tagliatelle by partially substituting a chickpea-teff formulation with carob flour (10-30%, w/w) and to investigate the relationships between carob bioactives and the physical, functional, and sensory properties of the resulting pasta.

**Methods:**

Tagliatelle samples containing different levels of carob flour were produced and characterized through physicochemical, technological, and sensory analyses. Phenolic compounds, antioxidant capacity, starch hydrolysis, and anti-inflammatory activity were evaluated. Oral processing behavior was assessed, and microstructural changes were examined by scanning electron microscopy. Consumer acceptance was tested in both plain and sauce-supplemented preparations.

**Results:**

Carob incorporation induced dose-dependent changes in the phenolic profile, which were associated with increased antioxidant capacity and reduced pro-inflammatory cytokine responses in a 2D intestinal mucosal co-culture model. D-pinitol and condensed tannins were associated with a reduction in starch hydrolysis, supporting a potential glycemic-modulating effect. Carob-enriched samples required a greater number of chews and generated harder, less adhesive boli, suggesting enhanced satiation potential. From a technological perspective, carob addition improved post-cooking firmness, water absorption, and swelling index, while scanning electron microscopy revealed the formation of a denser and more cohesive matrix. Sensory evaluation showed good overall acceptance, particularly among individuals with celiac disease.

**Discussion:**

Carob flour proved to be a technologically and functionally effective ingredient for gluten-free pasta formulation. A substitution level of 20% provided the best balance between technological performance, functional properties, and consumer acceptance. These findings support the use of carob flour as an evidence-based strategy for developing cereal-free gluten-free products with enhanced nutritional value and health-promoting potential.

## 1 Introduction

Pasta is one of the most widely consumed staple foods worldwide, valued for its versatility, affordability, and ease of preparation. However, traditional pasta is manufactured from wheat, making it unsuitable for individuals with gluten intolerance and celiac disease (1). This concern is particularly significant given that approximately 6% of the population in Europe and the United States is affected by non-celiac gluten sensitivity (2), while around 1% suffers from celiac disease (3). Beyond clinical necessity, gluten-free diets have gained considerable mainstream traction. A recent report by the Italian research institute Eurispes (4) estimated that nearly 21% of the population typically bases its diet on gluten-free products, reflecting a sustained and growing market expansion.

Despite the growing availability of gluten-free products in the market, these formulations remain widely criticized for their nutritional and technological shortcomings. Gluten-free formulations, typically based on rice and corn flour, are deficient in dietary fiber and lack a complete protein profile providing all essential amino acids (1). From a technological standpoint, the absence of gluten compromises structural cohesion and elasticity, resulting in excessive stickiness, adhesiveness, and reduced post-cooking firmness (5). Sensory quality is equally affected: gluten-free pasta frequently presents a dull, pale, or grayish appearance (6) and is perceived as bland or slightly bitter due to the absence of wheat’s natural sweetness (7), contributing to consistently low consumer acceptability (8).

Addressing these limitations requires the identification of alternative ingredients capable of simultaneously improving nutritional value, functional performance, and sensory quality. In this regard, chickpeas and teff have emerged as promising plant-based candidates. Chickpeas provide high-quality plant-based protein, essential amino acids, and dietary fiber, which contribute to gut health and satiety (9), while teff is notably rich in calcium, iron, magnesium, and resistant starch (10, 11). Nevertheless, incorporating these ingredients alone does not fully resolve the functional limitations of gluten-free matrices, as consumer demand increasingly extends beyond dietary compliance to include health-promoting properties such as antioxidant and anti-inflammatory activity and improved glycemic control (12, 13).

In this context, carob (*Ceratonia siliqua* L.) flour emerges as a particularly promising complementary ingredient. The elevated levels of polyphenols and dietary fiber present in the fruit are indicative of significant functional properties. In addition, the presence of bioactive compounds, such as D-pinitol, suggests a potential for therapeutic benefits, particularly in the regulation of blood glucose levels and the suppression of diabetes (14). From a technological perspective, caroubin, a structural protein native to carob, may contribute to reinforcing the integrity of gluten-free pasta matrices (15), and the ingredient’s natural sweetness has been shown to enhance the sensory profile of food products (16). Furthermore, as a traditional Mediterranean crop requiring minimal water and exhibiting natural pest resistance, carob offers meaningful sustainability advantages, supporting biodiversity and regional agricultural systems (17, 18).

Despite these attributes, the incorporation of carob flour into gluten-free pasta formulations remains largely unexplored. Most existing research has focused on bakery applications, leaving a significant knowledge gap regarding its technological, nutritional, and sensory effects specifically within pasta systems. The present study therefore aims to develop a novel gluten-free pasta enriched with carob flour and to evaluate its impact on nutritional composition, cooking quality, functional properties, and consumer sensory acceptability.

## 2 Materials and methods

### 2.1 Materials

Pepsin (P7012), pancreatin (P7545), porcine bile extract (B8631), aluminum chloride, *α*-amylase, amyloglucosidase, protease, lipopolysaccharide from *Escherichia coli* (LPS), and reagents for sugar and starch analysis (Luff-Schoorl solution, soluble starch, glucose, fructose, lactose, saccharose, maltose) were obtained from Merck KGaA (Germany). Reagents for antioxidant and polyphenol assays, 2,2-diphenyl-1-picrylhydrazyl (DPPH), Trolox, 2,2′-azobis(2-amidinopropane) dihydrochloride (AAPH), fluorescein, Folin–Ciocalteu, gallic acid, catechin, rutin, and vanillin, were purchased from Scharlab S. L. (Spain), along with general analytical reagents: acetonitrile, methanol, ethanol, pyridine, p-toluenesulfonyl chloride (TsCl), pentaerythritol, phosphoric, hydrochloric and sulfuric acids, sodium hydroxide, carbonate, bicarbonate, and chloride, potassium chloride and dihydrogen phosphate, magnesium chloride hexahydrate, calcium chloride dihydrate, ammonium carbonate, hexane, dimethyl sulfoxide (DMSO), sodium thiosulfate, potassium iodide, boric acid, and 3-(N-morpholino)propanesulfonic acid (MOPS) buffer.

### 2.2 Determination of the base pasta formulation

The study began by establishing a base gluten-free pasta formulation without carob (**F0**). Various combinations of flour (chickpea, teff, and corn), water content, and binding agents (egg and xanthan gum) were tested and evaluated for their suitability for lamination and cutting (Supplementary Table S1). Doughs were prepared using an electric mixer followed by manual mixing, stored at 4 °C, then sheeted and cut into tagliatelle (1 mm thick, 70 mm long) using a domestic pasta machine (19). Three independent batches of F0 were prepared and stored at 4 °C for no more than 24 h prior to analysis.

### 2.3 Enrichment of the base pasta with carob flour

Following establishment of F0, chickpea and teff flours were partially substituted with carob flour at levels of 10, 20, 25, and 30% (w/w), yielding formulations F10, F20, F25, and F30, respectively. The precise proportions of ingredients are provided in Supplementary Table S2.

### 2.4 Nutritional profiling

Protein, fat, and total dietary fiber were determined using AOAC (20) methods 920.87, 920.39, and 991.43, respectively. Starch was quantified after acid hydrolysis (3% HCl, v/v) using the Luff–Schoorl method (21). Simple sugars were separated and quantified by high-resolution anion-exchange chromatography (22), and total simple sugars calculated as the sum of individual sugars (23). Ash content was determined gravimetrically (24). Total carbohydrates were estimated as the sum of starch, simple sugars, and dietary fiber. All assays were performed in triplicate (mean ± SD, g/kg).

### 2.5 Physicochemical characterization

#### 2.5.1 Color

Color coordinates (L*, a*, b*) of uncooked and cooked pasta were measured in triplicate using a CR-5 spectrocolorimeter (Minolta, Japan; 10° observer, D65 illuminant). Chroma (C*ab), hue angle (h*ab), and total color difference (ΔE*) relative to **F0** were calculated using standard equations (25). Results are expressed as mean ± SD.

#### 2.5.2 Texture

Firmness of uncooked and cooked pasta was measured in triplicate using a TA. XT2 Texture Analyzer (Stable Micro Systems, UK; 25-kg load cell). Four samples were cut crosswise at 1 mm/s using a knife blade; firmness was recorded as the maximum cutting force (N) (19).

#### 2.5.3 Cooking quality: water absorption and swelling indices

Optimal cooking time was determined following AACC Method 16–50 (26) in triplicate. Briefly, 25 g of pasta was boiled in 250 mL deionized water at 98 °C for 2 min, drained, and cooking was stopped with cold water. Water absorption index (WAI, %) and swelling index (SI, %) were calculated as previously described (27), using the weights of uncooked and cooked pasta and their oven-dried counterparts (105 °C, constant weight).

#### 2.5.4 Microstructural characterization by CryoFESEM

Internal microstructure was analyzed by Cryo-Field Emission Scanning Electron Microscopy (CryoFESEM; ULTRA 55, Zeiss, Germany). Cooked tagliatelle cross-sections were sublimation-dried to preserve native structure (28), fixed on copper supports, platinum-coated, and observed at 3 kV. Micrographs were acquired at 300 × and 1,000 × magnifications to evaluate the organization of the matrix, starch granule integration, porosity, and the presence of fibrous structures.

#### 2.5.5 *In vivo* mastication and bolus texture analysis

Five healthy adult volunteers (aged 25–42 years) with complete dentition and no dental or temporomandibular disorders (29) chewed 5.0 ± 0.1 g of cooked tagliatelle until the natural swallowing threshold and expectorated the bolus. Chewing time (s) and number of chews were recorded. Bolus Texture Profile Analysis (TPA) was performed immediately after expectoration to preserve the native structure of the samples and minimize changes in textural properties prior to measurement. Five boluses per formulation were analyzed using a TA. XT2 analyzer (35-mm cylindrical probe; 70% compression, double cycle, 10 mm/s). Hardness (N), adhesiveness (N·s), and cohesiveness were recorded (Exponent software v6.1.11.0) (30).

### 2.6 *In vitro* digestion

*In vitro* digestion followed the standardized INFOGEST static protocol (31) with minor modifications. Samples were prepared by the chew-and-spit method (32). Volunteers chewed 5.0 ± 0.1 g of cooked pasta for 2 min, after which the expectorated bolus was collected. The bolus was adjusted to a final weight of 10 g by adding saliva from the same volunteer and was immediately kept on ice until the start of digestion. For the gastric phase, simulated gastric fluid and pepsin (2000 U/mL) were added (1:1, v/v); pH was adjusted to 3.0 with 6 M HCl; and the mixture was incubated at 37 °C for 120 min. For the intestinal phase, simulated intestinal fluid, pancreatin (100 U/mL trypsin activity), and bile salts (10 mM) were added (1:2, v/v); pH was adjusted to 7.0 with 1 M NaOH; and the mixture was incubated for 120 min. Proteolysis was stopped with Pefabloc (1 μL, 500 mM). After centrifugation (8,000 × g, 10 min, 4 °C), supernatants were stored at −20 °C. Three independent *in vitro* digestions were performed for each formulation, with blank samples and undigested controls included in each run.

### 2.7 Quantification of phenolic compounds

#### 2.7.1 Total phenolic content

Total phenolic content (TPC) was determined using the Folin–Ciocalteu method (33). Absorbance was read at 765 nm (VANTAstar, BMG Labtech, Germany) and results expressed as mg gallic acid equivalents (GAE)/kg digested pasta (mean ± SD, triplicate).

#### 2.7.2 Total flavonoid content

Total flavonoid content (TFC) was determined by AlCl₃ colorimetry (34). Absorbance was read at 415 nm and results expressed as mg rutin equivalents (RE)/kg digested pasta (mean ± SD, triplicate).

#### 2.7.3 Condensed tannins

Condensed tannins were quantified using the acidified vanillin method (35) with modifications. Absorbance was read at 500 nm and results expressed as mg catechin equivalents (CE)/kg digested pasta (mean ± SD, triplicate).

### 2.8 D-pinitol quantification

D-pinitol was quantified by HPLC-UV following the method proposed by Pereira et al. (36) with minor modifications. Lyophilized samples (72 h) were derivatized with TsCl in pyridine at 50 °C for 3 h. Pentaerythritol was used as an internal standard. Samples then analyzed on a Hitachi LaChrom Elite HPLC system with an InfinityLab Poroshell 120 EC-C18 column (Agilent, Spain; detection at 230 nm). Results are expressed as mg D-pinitol/kg digested pasta (mean ± SD, triplicate).

### 2.9 Starch digestibility

Starch digestibility of digested formulations was assessed by quantifying reducing sugar release using the 3,5-dinitrosalicylic acid (DNS) method (37). White bread was included as a reference food. Results are expressed as g glucose equivalents/kg digested cooked sample (mean ± SD, triplicate).

### 2.10 Antioxidant activity

Antioxidant activity was assessed by DPPH and ORAC assays (38). For DPPH, absorbance was measured at 517 nm after 60 min incubation in the dark (33). For ORAC, fluorescein decay (Ex/Em: 485/538 nm) was recorded every minute for 100 min after AAPH addition (39). All measurements were performed in triplicate using a VANTAstar microplate reader; Trolox (2–104 μM) was used for calibration. Results are expressed as mg Trolox equivalents (TE)/kg digested pasta.

### 2.11 Anti-inflammatory activity

HT-29 and Caco-2 cells (Cytion, Germany) were cultured in Dulbecco’s Modified Eagle Medium (DMEM) supplemented with 10% heat-inactivated Fetal Calf Serum (FCS), 2 mM L-glutamine, and 1% penicillin–streptomycin (Merck, Spain) at 37 °C, 5% CO₂. Cytocompatibility was assessed following Crisafulli et al. (40) with minor modifications: Caco-2: HT-29 cells (3:1) were seeded at 1 × 10^5^ cells/well, treated with pasta extracts (0.2–1.0 mg/mL) for 48 h, and cell viability assessed using PrestoBlue™.

Anti-inflammatory activity was assessed following Toshina et al. (41) with modifications. A Caco-2: HT-29 co-culture (3:1) was maintained for 21 days to establish a 2D mucus-like barrier. LPS (10 ng/mL) was added for 24 h to induce inflammation, cells were subsequently washed with PBS and treated with pasta samples (1 mg/mL). Concentrations of IL-1β, IL-6, and TNF-*α* in collected supernatants were determined using ELISA kits (Abcam, Netherlands). Results are expressed as mean ± SD from triplicate experiments.

### 2.12 Sensory analysis

Sensory evaluation was conducted in individual booths compliant with UNE-EN ISO 8589, 2010 (42). Participants completed a questionnaire including sex, age, pasta consumption frequency, and familiarity with carob as a food ingredient. Overall acceptance assessed using a 9-point hedonic scale (43), and color, odor, texture, and flavor on a 5-point just-about-right (JAR) scale (score of 3 = optimal). Purchase intention was assessed on a 5-point scale. The evaluation was conducted over two sessions. In the first session, 70 non-celiac participants assessed all formulations (F0–F30) served plain. Based on results, the lowest-rated formulation was excluded from the second session. In the second session, 20 celiac participants evaluated selected formulations both plain and with commercial tomato sauce. Given the need to evaluate both serving conditions in a single session, the number of formulations was reduced, excluding **F30** due to its lack of significant differences in the previous test. Most celiac participants were recruited through the *Asociación de Celíacos de la Comunidad Valenciana*.

### 2.13 Statistical analysis

One-way ANOVA followed by Fisher’s LSD test assessed differences in nutritional, technological, and functional parameters among formulations (*p* < 0.05; Statgraphics Centurion XIX). Sensory data were analyzed with XLSTAT 2020 (Addinsoft). Kruskal–Wallis tests followed by Dunn’s *post hoc* test with Bonferroni correction (*p* < 0.05) were applied to acceptance and purchase intention data. Penalty analysis was performed using overall acceptance and JAR data (44); the 5-point JAR scale was simplified to 3 points (45), and ANOVA with Tukey’s HSD test was applied.

### 2.14 Ethical considerations

All human subject procedures were conducted in accordance with the Declaration of Helsinki. Participants were informed about the study purpose and provided consent before participating. The study protocol was approved by the Ethics Committee of the Universitat Politècnica de València (Reference Number: P01_30-06-2025).

## 3 Results

### 3.1 Determination of the fresh pasta formulation

The lamination and cutting performance of various gluten-free formulations (Supplementary Table S1) was systematically evaluated to identify the most suitable base dough. Among those tested, only formulation 6 exhibited adequate mechanical properties for successful lamination and cutting. This optimal base formulation (338 g/kg each of chickpea and teff flour, 14 g/kg xanthan gum, and 311 g/kg g water) was designated as F0. Carob flour was subsequently incorporated by substituting the chickpea-teff blend at 10, 20, 25, and 30%, yielding formulations F10, F20, F25, and F30. Precise ingredient proportions are provided in Supplementary Table S2.

### 3.2 Nutritional profile

Table 1 presents the nutritional composition of the gluten-free tagliatelle formulations. Protein and fat content decreased significantly with increasing carob substitution (*p* < 0.05), showing reductions of approximately 20 and 37% from F0 to F30, respectively. Carob enrichment led to a slight increase in total carbohydrate content (ca. 5%; *p* < 0.05), which remained the predominant macronutrient across all formulations. Simple sugar content increased markedly by approximately 183%, while starch content decreased by around 23%. Total dietary fiber showed a substantial increase of ca. 51%. No significant differences in ash content were observed among formulations (*p* > 0.05).

**Table 1:** Nutritional composition (mean ± SD, *n* = 3) of gluten-free pasta formulations (g/kg fresh weight).

**Nutrient (g/kg)**	**F0**	**F10**	**F20**	**F25**	**F30**
Protein	91 ± 2^a^	86 ± 1^b^	80 ± 3^c^	76 ± 2^d^	73 ± 1^e^
Fat	27 ± 3^a^	23 ± 1^b^	21 ± 2^c^	19 ± 1^cd^	17 ± 2^d^
Total carbohydrates	531 ± 5^a^	540 ± 3^b^	540 ± 5^b^	550 ± 3^bc^	557 ± 1^c^
Simple sugars	24 ± 1^e^	41 ± 1^d^	54 ± 1^c^	62 ± 2^b^	68 ± 2^a^
Starch	372 ± 7^a^	346 ± 9^b^	313 ± 6^c^	302 ± 6^d^	285 ± 5^e^
Total dietary fiber	135 ± 6^d^	153 ± 9^c^	173 ± 16^b^	186 ± 9^ab^	204 ± 17^a^
Ash	14.2 ± 0.1	14.3 ± 0.0	14.5 ± 0.2	14.6 ± 0.1	14.5 ± 0.2

Formulations: **F0** (chickpea:teff:carob = 50:50:0); **F10** (45:45:10); **F20** (40:40:20); **F25** (37.5:37.5:25); **F30** (35:35:30). Different lowercase letters within the same row indicate significant differences among formulations (*p* < 0.05).

### 3.3 Physicochemical characterization

#### 3.3.1 Color

Table 2 shows the color parameters and total color differences (ΔE*) of uncooked and cooked tagliatelle. F0 displayed a vivid yellow hue attributable to carotenoid pigments in the base flours. Progressive carob flour addition significantly decreased L*, C*ab, and h*ab (*p* < 0.05), shifting color toward darker, reddish-brown tones. Despite cooking, ΔE* values remained broadly similar across formulations, indicating color stability throughout the cooking process.

**Table 2:** Color parameters (L*, C*ab, h*ab) and total color difference (ΔE*) relative to **F0** for uncooked and cooked tagliatelle (mean ± SD, *n* = 3).

	**Uncooked**				**Cooked**			
**Formulation**	L*	C*ab	h*ab	ΔE*	L*	C*ab	h*ab	ΔE*
F0	53.10 ± 2.68^a^	21.07 ± 2.15^aA^	74.49 ± 1.57^aB^	—	54.52 ± 0.90^a^	17.78 ± 1.79^aB^	84.27 ± 1.60^aA^	—
F10	36.00 ± 2.6^9b^	13.65 ± 1.30^bB^	60.04 ± 1.91^bB^	19.19 ± 2.30^d^	36.96 ± 1.43^b^	17.53 ± 0.16^aA^	63.43 ± 1.24^bA^	18.68 ± 1.47^c^
F20	32.22 ± 1.26^cA^	10.22 ± 1.27^cB^	56.60 ± 2.42^bc^	23.99 ± 1.11^cB^	28.92 ± 1.89^cB^	13.81 ± 2.62^bA^	56.84 ± 2.13^c^	26.92 ± 1.99^bA^
F25	29.91 ± 2.66^cdA^	6.30 ± 0.63^dB^	57.92 ± 1.97^bcA^	27.72 ± 2.11^bB^	24.67 ± 1.81^dB^	9.14 ± 1.78^cA^	54.81 ± 1.85^cdB^	31.73 ± 1.45^aA^
F30	27.38 ± 1.03d^A^	6.42 ± 0.84^d^	55.59 ± 3.55^c^	30.30 ± 1.16^aB^	23.42 ± 0.92^dB^	5.43 ± 1.82^c^	52.62 ± 2.22^d^	33.48 ± 0.91^aA^

Formulations: **F0** (50:50:0); **F10** (45:45:10); **F20** (40:40:20); **F25** (37.5:37.5:25); **F30** (35:35:30). L*, lightness; C*ab, chroma; h*ab, hue angle; ΔE*, total color difference relative to F0. Different lowercase letters within the same column indicate significant differences among formulations; different uppercase letters within the same row indicate significant differences between uncooked and cooked samples (*p* < 0.05). Absence of uppercase letters indicates non-significant differences.

#### 3.3.2 Texture and cooking quality indices

Table 3 shows firmness values and cooking quality indices. No significant firmness differences were observed among uncooked formulations (*p* > 0.05). After cooking, F0 and F10 showed the lowest firmness (3.08 and 2.92 N, respectively), while F20, F25, and F30 exhibited significantly higher values (4.32–4.83 N) (*p* < 0.05). Carob flour significantly increased both WAI and SI (*p* < 0.05). No significant differences between F20, F25, and F30 were observed for these parameters (*p* > 0.05).

**Table 3:** Firmness (N), WAI (%), and SI (%) of tagliatelle (mean ± SD, *n* = 3).

**Parameter**	**F0**	**F10**	**F20**	**F25**	**F30**
Firmness – Uncooked (N)	12.43 ± 1.8	12.61 ± 1.2	12.91 ± 0.4	13.34 ± 0.9	14.10 ± 1.8
Firmness – Cooked (N)	3.08 ± 0.53^a^	2.92 ± 0.55^a^	4.32 ± 1.29^b^	4.66 ± 0.32^b^	4.83 ± 0.60^b^
WAI (%)	79.7 ± 4.7^a^	79.7 ± 4.7^a^	95.1 ± 5.1^b^	102.4 ± 7.3^c^	106.9 ± 6.9^c^
SI (%)	42.5 ± 0.2^a^	43.1 ± 0.1^a^	46.5 ± 0.3^b^	46.7 ± 0.1^b^	46.9 ± 0.2^b^

Formulations: **F0** (50:50:0); **F10** (45:45:10); **F20** (40:40:20); **F25** (37.5:37.5:25); **F30** (35:35:30). Lowercase letters within the same row indicate significant differences among formulations (*p* < 0.05). Absence of lowercase letters indicates non-significant differences.

#### 3.3.3 Microstructural characterization

**Figure 1:**
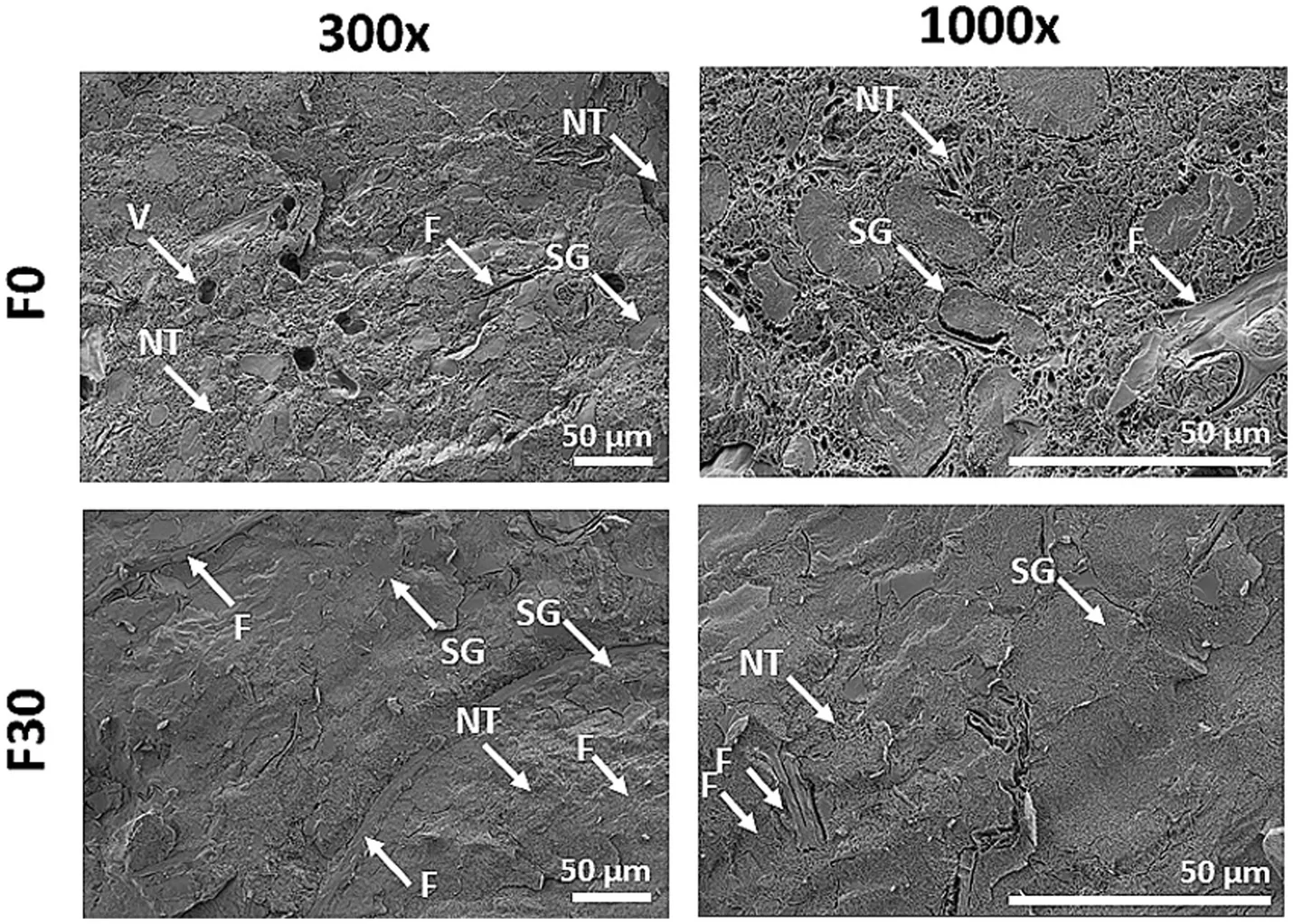
CryoFESEM micrographs of F0 and F30 fresh tagliatelle at 300 × and 1,000 × magnification. F, dietary fiber; NM, network matrix; SG, starch granules; V, voids. Chickpea:teff:carob ratios: F0 = 50:50:0; F30 = 35:35:30.

Figure 1 exhibits CryoFESEM micrographs of cooked F0 and F30 at 300 × and 1,000 × magnification. In F0, multiple voids (V) were observed, and the network matrix (NM) appeared highly porous with numerous visible gaps. Starch granules (SG) were mostly intact but amorphous, with large white spaces surrounding them indicating poor matrix integration. In contrast, F30 displayed a more cohesive and organized microstructure with virtual absence of voids, a denser and more continuous network matrix, smaller and more uniformly distributed pores, clearly visible fibrous-like structures (F), which may be associated with the incorporation of carob flour. No cracks or structural discontinuities were observed.

### 3.4 *In vivo* bolus texture analysis

Table 4 indicates chewing behavior and TPA parameters of cooked, masticated pasta. The number of chews increased by approximately 56% and chewing time by approximately 40% from F0 to F30 (*p* < 0.05). Bolus hardness increased from 30.88 N (F0) to 63.91 N (F30) (*p* < 0.05). Cohesiveness was not significantly affected by carob addition (*p* > 0.05). Adhesiveness decreased significantly with carob enrichment, from −0.47 N·s (F0) to −0.12 N·s (F10) (*p* < 0.05), with no significant differences observed among F20, F25, and F30.

**Table 4:** Number of chews, chewing time (s), and bolus TPA parameters [hardness (N), cohesiveness, and adhesiveness (N·s)] of cooked and masticated tagliatelle (mean ± SD, *n* = 5).

**Parameter**	**F0**	**F10**	**F20**	**F25**	**F30**
Number of chews	16 ± 3^a^	19 ± 3^ab^	22 ± 4^b^	22 ± 4^b^	25 ± 4^c^
Chewing time (s)	15 ± 3^a^	17 ± 4^ab^	19 ± 5^b^	19 ± 5^b^	21 ± 4^b^
Hardness (N)	30.88 ± 7.10^a^	33.01 ± 5.33^a^	47.73 ± 6.17^b^	49.66 ± 6.78^b^	63.91 ± 6.40^c^
Cohesiveness (%)	0.24 ± 0.05	0.22 ± 0.03	0.23 ± 0.01	0.26 ± 0.03	0.26 ± 0.02
Adhesiveness (N·s)	−0.47 ± 0.12^c^	−0.12 ± 0.01^a^	−0.18 ± 0.08^b^	−0.22 ± 0.07^b^	−0.29 ± 0.05^b^

Formulations: **F0** (50:50:0); **F10** (45:45:10); **F20** (40:40:20); **F25** (37.5:37.5:25); **F30** (35:35:30). Different lowercase letters within the same row indicate significant differences among formulations (*p* < 0.05). Absence of lowercase letters indicates non-significant differences.

### 3.5 Bioactive compounds and functional properties after *in vitro* digestion

#### 3.5.1 Phenolic compounds, antioxidant activity, D-Pinitol, and starch digestibility

Table 5 shows the bioactive compound content and functional properties of digested formulations. Total polyphenols increased by 67%, total flavonoids by 223%, and condensed tannins by 219% from F0 to F30 (*p* < 0.05). DPPH antioxidant activity increased by 496% and ORAC by 63% across the same range. D-pinitol was absent in F0 and increased dose-dependently with carob content, reaching 36.2 mg/kg in F30 (*p* < 0.05). Starch digestibility decreased progressively with increasing carob substitution, from 336.2 g glucose equivalents/kg g in F0 to 243 g/kg g in F30. Both F0 and F30 yielded lower starch digestibility than white bread (413 g glucose equivalents/kg), by 19 and 42%, respectively.

**Table 5:** Bioactive compound content and functional properties of digested tagliatelle formulations (mean ± SD, *n* = 3). TPC, total polyphenol content (mg GAE/kg); TFC, total flavonoid content (mg RE/kg); tannins (mg CE/kg); DPPH and ORAC antioxidant activity (mg TE/kg); D-pinitol (mg/kg); starch digestibility (g glucose equivalents/kg).

**Parameter**	**F0**	**F10**	**F20**	**F25**	**F30**
Total polyphenols (mg GAE/kg)	1,400 ± 21^a^	1706 ± 32^b^	2090 ± 70^c^	2,250 ± 50^d^	2,340 ± 70^d^
Total flavonoids (mg RE/kg)	267 ± 36^a^	356 ± 25^b^	552 ± 23^c^	718 ± 53^d^	863 ± 4^e^
Total tannins (mg CE/kg)	99 ± 30^a^	160 ± 15^b^	235 ± 5^c^	256 ± 13^c^	316 ± 56^d^
DPPH (mg TE/kg)	302 ± 20^a^	810 ± 75^b^	1,380 ± 150^c^	1,610 ± 200^d^	1800 ± 190^d^
ORAC (mg TE/kg)	6,480 ± 170^a^	7,070 ± 270^b^	8,510 ± 90^c^	9,850 ± 180^d^	10,550 ± 110^e^
D-Pinitol (mg/kg)	n.d.^a^	11.3 ± 1.3^b^	29.7 ± 1.5^c^	31.8 ± 0.7^d^	36.2 ± 1.5^d^
Starch digestibility (g glc eq./kg)	336.2 ± 5^a^	304.2 ± 7^b^	267.5 ± 5.3^c^	257 ± 4^cd^	243 ± 6^d^

Formulations: **F0** (50:50:0); **F10** (45:45:10); **F20** (40:40:20); **F25** (37.5:37.5:25); **F30** (35:35:30). n.d., not detected. GAE, gallic acid equivalents; RE, rutin equivalents; CE, catechin equivalents; TE, Trolox equivalents. Different lowercase letters within the same row indicate significant differences among formulations (*p* < 0.05).

#### 3.5.2 Anti-inflammatory activity in a Caco-2: HT-29 co-culture model

**Figure 2:**
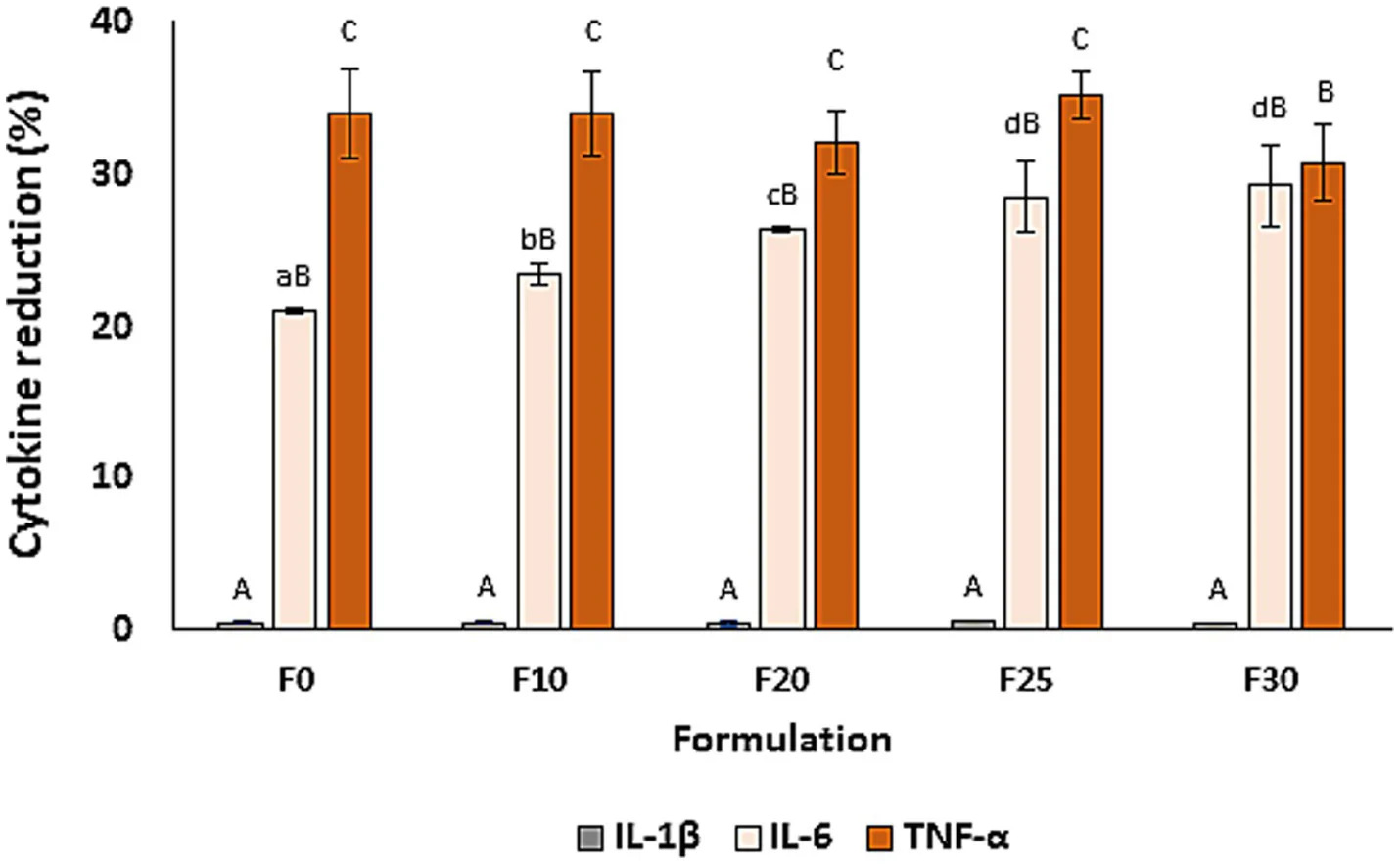
Percentage inhibition of pro-inflammatory cytokines (IL-1β, IL-6, and TNF-*α*) in the Caco-2: HT-29 co-culture model following exposure to digested pasta formulations. Data are presented as mean ± *SD* (*n* = 3). Different lowercase letters indicate statistically significant differences between pasta samples; different uppercase letters denote significant differences between cytokines. Formulations: **F0** (50:50:0); **F10** (45:45:10); **F20** (40:40:20); F25 (37.5:37.5:25); **F30** (35:35:30).

Cytokine patterns following LPS administration are presented in Figure 2. IL-1β was undetectable under all conditions. Both IL-6 and TNF-*α* were detectable after 24 h of LPS stimulation. TNF-α showed an approximate 35% reduction following exposure to digested pasta across all formulations, with no significant differences among them (*p* > 0.05). For IL-6, F0 reduced its production by approximately 20%, whereas F30 resulted in approximately 30% suppression (*p* < 0.05).

### 3.6 Sensory analysis

As detailed in Supplementary Table S3, the panel comprised 70 non-celiac and 20 celiac participants, most of whom were regular pasta consumers. Familiarity with carob as a food ingredient was reported by 22% of non-celiac and 45% of celiac participants.

**Figure 3:**
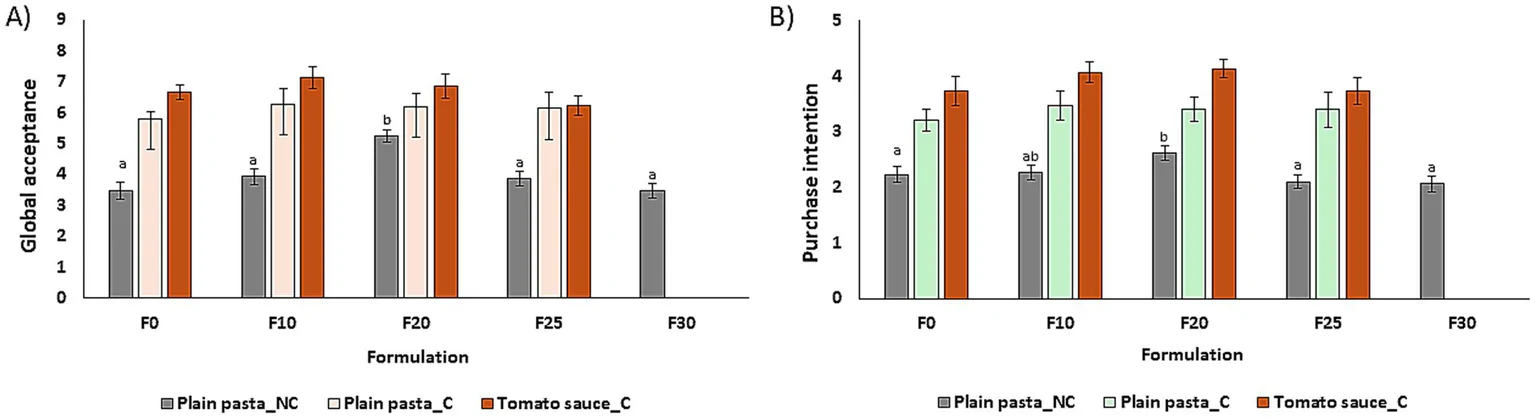
Overall acceptance **(A)** and purchase intention **(B)** for tagliatelle formulated with different chickpea:teff:carob ratios: **F0** (50:50:0); **F10** (45:45:10); **F20** (40:40:20); **F25** (37.5:37.5:25); **F30** (35:35:30). NC, non-celiac participants; C, celiac participants; Tomato sauce_C, celiac participants with tomato sauce. Lowercase letters indicate significant differences between formulations (*p* < 0.05).

Among non-celiac participants, significant differences were found in overall acceptance and purchase intention (*p* < 0.05). Carob substitution up to 20% (F20) improved acceptance relative to F0, while F25 and F30 yielded scores similar to F0 (Figure 3). Celiac participants showed no significant differences between formulations in acceptance (5.8–6.3 out of 9) or purchase intention (3.2–3.4 out of 5) (*p* > 0.05), and rated all formulations approximately 3 and 1 points higher than non-celiac participants on these respective scales. When celiac participants evaluated the same formulations with tomato sauce, acceptance scores reached 6.7–7.1 and purchase intention 3.7–4.1, with no significant differences between formulations (*p* > 0.05).

JAR analysis (Supplementary Figures S1A–C) identified color (perceived as too dark) and flavor (perceived as too intense) as the least optimized attributes in plain carob-enriched pasta. These deviations were effectively masked by tomato sauce addition.

**Figure 4:**
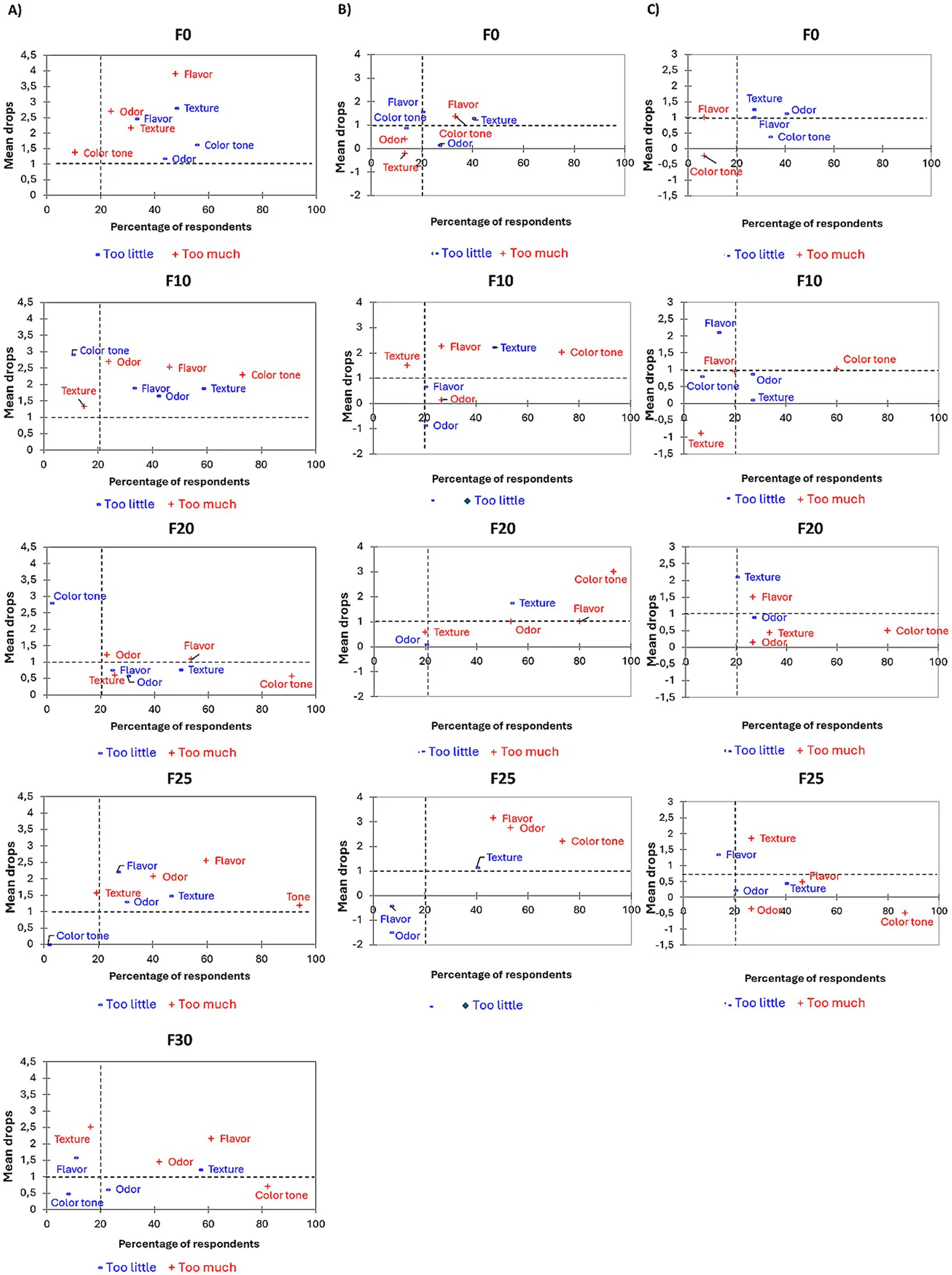
Penalty analysis showing mean drops in overall acceptance as a function of the proportion of participants who perceived each sensory attribute as deviating from ideal (“too little,” blue; “too much,” red). **(A)** Plain pasta by non-celiac participants; **(B)** Plain pasta by celiac participants; **(C)** Pasta with tomato sauce by celiac participants. Formulations: **F0** (50:50:0); **F10** (45:45:10); **F20** (40:40:20); **F25** (37.5:37.5:25); **F30** (35:35:30).

Penalty analysis results are shown in Figure 4. For non-celiac participants (Figure 4A), F0 showed significant drops due to excessively intense flavor and odor and overly soft texture. F10 reduced these penalties, and at F20 only intense odor remained a significant penalty (*p* < 0.05). At F25 and F30, mean drops increased again due to excessive odor and flavor intensity (*p* < 0.05). For celiac participants evaluating plain pasta (Figure 4B), flavor, color, and texture caused modest drops for F0; these penalties diminished progressively up to F20. At F25, color, odor, and flavor all contributed significantly to reduced acceptance (*p* < 0.05). Penalty analysis of the product with tomato sauce (Figure 4C) revealed a masking effect. In fact, sensory attributes expected to strongly influence overall acceptability following carob enrichment, such as color and flavor, were not significant in F25.

## 4 Discussion

The results obtained across all substitution levels are presented and discussed in two thematic sections. The first addresses the changes in nutritional composition and functional properties induced by carob enrichment, including proximate composition, bioactive compound content, antioxidant capacity, starch digestibility, and anti-inflammatory activity. The second examines the physicochemical and technological behavior of the formulations (encompassing color, cooking quality, microstructure and oral processing) and discusses how these changes translate into sensory-relevant attributes. Together, both perspectives provide an integrated view of the suitability of carob flour as a functional ingredient in gluten-free pasta systems.

### 4.1 Nutritional and functional analysis

The progressive decrease in protein and fat content with increasing carob substitution reflects the lower protein (ca. 4%) and fat (ca. 1%) content of carob flour compared with chickpea and teff flours. While this represents a nutritional trade-off, the compensating increase of 1.5-fold in total dietary fiber is of considerable nutritional significance. A similar increase in dietary fiber following carob incorporation was previously reported by Arribas et al. (46) in rice-white bean gluten-free fettuccine fortified with carob fruit flour, confirming the contribution of carob as an effective fiber-enriching ingredient in gluten-free pasta systems. Higher fiber content may enhance satiety by slowing gastric emptying (47) and supports gastrointestinal health by improving intestinal transit and promoting microbial diversity (48). The insoluble fiber fraction, which constitutes approximately 90% of total fiber in carob flour (49), likely plays a key role in stimulating short-chain fatty acid (SCFA) production and in improving bioavailability of polyphenols through microbial fermentation (50). SCFAs support colonocyte function and contribute to systemic metabolic regulation (48). The increase in simple sugars attributable to the naturally high sucrose content of carob flour, was offset by a decrease in starch from ca. 23%, thereby reducing the availability of D-glucose for intestinal absorption. This is particularly relevant for individuals with diabetes or impaired glucose tolerance. Although no significant differences in ash content were observed, carob is naturally rich in potassium and calcium, which may contribute to cardiovascular, musculoskeletal, and metabolic health (17).

The increase in polyphenols, flavonoids, and condensed tannins across formulations highlight carob as a rich source of bioactive compounds with known antioxidant, anti-inflammatory, and enzyme-inhibitory activities (17, 51, 52). The markedly greater increase in DPPH (ca. 500%) compared to ORAC (ca. 63%) likely reflects the high efficiency of condensed tannins and other high-molecular-weight polyphenols as electron donors, which increase with carob enrichment. This mechanism is preferentially captured by the DPPH assay, whereas ORAC primarily assesses hydrogen atom transfer capacity. Overall, the enhancement observed in both assays suggests an improved antioxidant potential, which may contribute to mitigating oxidative stress, a key mechanism underlying chronic diseases such as cardiovascular disorders and metabolic conditions (53, 54).

The dose-dependent accumulation of D-pinitol, absent in F0, indicates that carob is the sole source of this cyclitol in the formulation and suggests its stability under digestive conditions. Among carob bioactives, D-pinitol plays a prominent role due to its insulin-mimetic properties, promoting glucose uptake and modulating insulin signaling (55). On the other hand, the progressive reduction in starch digestibility is attributable to three synergistic mechanisms: (i) lower total D-glucose content available for hydrolysis; (ii) elevated dietary fiber acting as a physical barrier limiting enzyme access to starch (56); and (iii) polyphenols inhibiting digestive enzymes such as *α*-amylase and α-glucosidase (57). The combination of slower starch digestion and D-pinitol’s insulin-mimetic effects (58) suggests that carob-enriched gluten-free pasta could attenuate postprandial glycemic responses, a property particularly relevant for individuals with type 2 diabetes or those seeking improved glycemic control and satiety (59).

The evaluation of anti-inflammatory properties is particularly relevant in the context of gastrointestinal disorders associated with chronic mucosal inflammation, such as Crohn’s disease and ulcerative colitis, where cytokine overproduction compromises epithelial barrier integrity (60, 61). In our study, carob-enriched pasta after *in vitro* digestion revealed cytokine-specific responses. The lack of detectable IL-1β across all conditions is consistent with the established resistance of this cytokine to induction by LPS alone in intestinal epithelial cell lines (62). The dose-dependent suppression of IL-6 aligns with the well-known anti-inflammatory effects of polyphenols, particularly flavonoids, and dietary fiber (61, 63), as well as with the observed increase in these components following carob enrichment of the pasta (Section 3.5.1). The absence of significant differences in TNF-*α* suppression among formulations may reflect a combined anti-inflammatory contribution from all flour components, consistent with previously reported activities of chickpea and teff (64, 65).

Collectively, carob flour incorporation induces a coherent and nutritionally meaningful shift in gluten-free pasta, integrating nutritional and functional improvements into a unified health-oriented profile. While modest reductions in protein and fat were observed, these were effectively compensated by a substantial increase in dietary fiber and a reconfiguration of carbohydrate composition, collectively contributing to reduced glucose availability and potential glycemic attenuation. Functionally, the enrichment in phenolic compounds translated into enhanced antioxidant capacity and selective anti-inflammatory effects, particularly reflected in the suppression of IL-6. In parallel, the exclusive contribution of D-pinitol by carob, together with reduced starch digestibility, supports a synergistic mechanism targeting postprandial metabolic regulation. Overall, the nutritional and functional results position gluten-free pasta enriched with carob as a promising functional food, with potential benefits for people suffering from metabolic and inflammatory disorders. These findings suggest that the compositional changes induced by carob incorporation are closely linked to the observed biological responses, underscoring the close relationship between ingredient composition and functional performance in gluten-free pasta systems.

### 4.2 Physicochemical, oral processing and sensory analysis

In terms of color, the progressive darkening and shift toward reddish-brown tones with increasing carob content reflects the contribution of carob pigments, phenolic-protein interactions, and mild oxidation reactions (17, 66). The color shift is consistent with previous reports on pasta or in other gluten-free products enriched with carob (15, 46, 67). The notable stability of ΔE* between uncooked and cooked states is likely attributable to the short cooking time, limited pigment leaching, or sample uniformity (68). This is particularly advantageous, since gluten-free pasta often exhibits a pale, unappealing appearance, whereas formulations containing carob retained a vivid, warm color that may positively influence consumer perception (15).

The lack of significant differences in uncooked firmness suggests that carob flour does not substantially alter dough mechanical properties prior to cooking. However, upon thermal processing, carob incorporation led to a marked increase in firmness at ≥20% substitution (4.32–4.83 N vs. 3.08 N for **F0**), most likely attributable to the higher fiber content reinforcing structural integrity and resistance to deformation (69). Similar increases in cooked pasta firmness have been reported in carob-enriched gluten-free fettuccine, suggesting that the technological benefits associated with carob incorporation may be maintained across different gluten-free matrices despite substantial differences in formulation composition (46). This behavior is consistent with the observed increases in WAI and SI, which reflect the enhanced hydration and network-forming ability of carob polysaccharides during thermal processing. These properties arise from their hydrophilic nature and high hydroxyl group density, which promote hydrogen bonding with water (70–72). Accordingly, Hallaç and Altıner (73) reported a WAI capacity of 185.5% for carob flour compared to 104.7% for wheat flour. The plateau observed at ≥20% substitution (no significant differences between F20, F25, and F30) suggests that, at higher inclusion levels, competition for water between fiber and starch limits further increases in hydration capacity (72, 74).

The CryoFESEM findings confirm the structural reinforcement attributable to carob flour and are consistent with the texture and cooking quality results. The highly porous, void-rich matrix observed in F0 reflects a weaker network typical of gluten-free pasta lacking viscoelastic reinforcement (75). Poor starch granule integration in the base formulation further supports this interpretation and has been previously linked to reduced structural cohesion in composite flour systems (76). In contrast, the improved matrix cohesion in F30 indicates that carob fiber promotes fiber–protein–starch interactions that reinforce structural integrity, limit water migration during cooking, and preserve pasta structure (74). The presence of fibrous structures further supports the role of carob-derived polysaccharides in network formation.

Oral processing analysis demonstrated that mastication changed significantly with carob enrichment, with progressive increases in both chew count (ca. 55%) and chewing time (ca. 40%) from F0 to F30, parameters closely associated with increased satiety. This feature could be particularly beneficial in the dietary management of type 2 diabetes and obesity (19). Three mechanisms likely contribute: increased WAI enlarges sample volume during oral processing (Section 3.3.2); higher post-cooking firmness requires more chewing cycles to achieve a swallowable bolus (37); and a more cohesive protein-starch matrix demands greater mechanical effort (Section 3.3.3). The increase in bolus hardness is consistent with the higher insoluble fiber fraction, which is more resistant to mechanical disruption (77, 78), and corroborates the higher post-cooking firmness values (79). The significant reduction in bolus adhesiveness with carob addition is particularly relevant, as stickiness is a common complaint in gluten-free pasta consumption and affects both mouthfeel and handling during cooking (80, 81).

Regarding the sensory analysis of the developed tagliatelle, among non-celiac participants, the improvement in acceptance from F0 to F20 and subsequent decline at higher substitution levels is consistent with a masking effect of carob’s sweet, aromatic profile (80) on the unpleasant nutty and starchy notes of chickpeas (81) and polyphenol-mediated modulation of taste perception (82). The penalties observed at F25 and F30 are attributable to the astringent and slightly bitter taste of carob at higher concentrations (83), indicating a dose-dependent sensory threshold. The significantly lower adhesiveness of carob-enriched boluses (Section 3.4) aligns with the reduced ‘too soft texture’ observed in the penalty analysis, confirming the positive contribution of carob to mouthfeel. These findings agree with those reported by Arribas et al. (46), who observed good consumer acceptance of gluten-free fettuccine enriched with carob flour. Collectively, these results suggest that moderate levels of carob incorporation can improve consumer perception of gluten-free pasta, whereas excessive inclusion may negatively affect sensory acceptance due to the increased intensity of characteristic carob-derived flavor notes.

The higher acceptance scores among celiac compared with non-celiac participants (approximately 3 points on the 9-point scale) may be attributed to greater familiarity with gluten-free product characteristics, adjusted sensory expectations (84, 85), higher prior awareness of carob (45% vs. 22%), and the motivational value of products meeting dietary restrictions (86). This finding has practical implications, reinforcing the market viability of carob-enriched gluten-free pasta in the target consumer segment.

The effectiveness of tomato sauce in masking color and flavor deviations explains the pronounced improvement in acceptance and purchase intention scores observed when tomato sauce was included, and confirms that F20 is the optimal substitution level under realistic consumption conditions as no sensory attribute caused a significant penalty drop in this formulation when served with sauce (Figure 4C).

Bearing this in mind, carob incorporation was associated with progressive improvements in the structural organization of the gluten-free pasta matrix, which was consistently reflected in sensory perception. The formation of a more compact and cohesive fiber-protein-starch network, as suggested by increased post-cooking firmness, higher water absorption, and microstructural features observed by Cryo-FESEM, corresponded with reduced bolus adhesiveness and improved mouthfeel, addressing a common textural limitation of gluten-free pasta. In line with Ribes et al. (87), these properties may also favor more effective oral processing, potentially contributing to enhanced satiety and facilitating safe bolus formation. Furthermore, these physicochemical improvements were reflected in higher consumer acceptance, particularly at 20% substitution, where carob’s natural sweetness masked undesirable base-flour notes without inducing the astringency observed at higher levels. This trend was further reinforced when the pasta was served with tomato sauce.

## 5 Conclusion

This study demonstrates that partial substitution of the chickpea-teff base with carob flour at 20% (w/w) represents an optimal formulation for developing nutritionally and functionally enhanced gluten-free pasta.

Carob enrichment significantly increased dietary fiber content and introduced D-pinitol, with relevant implications for glycemic control. Functionally, carob-enriched formulations exhibited higher antioxidant capacity and reduced IL-6 production in an intestinal mucosal model, supporting a link between carob bioactives and anti-inflammatory activity. Structurally, CryoFESEM analysis revealed a denser and more cohesive protein-starch-fiber matrix, which was associated with improved cooking firmness, water retention, and oral processing behavior, suggesting enhanced satiation potential.

Finally, sensory evaluation confirmed good overall acceptability among celiac consumers, particularly when served with tomato sauce, highlighting the feasibility of developing carob-enriched pasta with improved functional properties without compromising consumer acceptance or market potential.

Although this work provides a solid scientific basis for the design of sustainable functional gluten-free pasta products targeting celiac and health-conscious consumers, future studies should investigate the *in vivo* bioavailability of bioactive compounds to confirm their physiological relevance and health benefits.

## Data Availability

The raw data generated in this study are available in the Zenodo repository (https://zenodo.org) under the doi: 10.5281/zenodo.20645046.
