# Supplementary material for: Carob-enriched gluten-free pasta: a sustainable approach for enhancing nutritional, functional, and sensory properties
Source: Front Nutr. 2026 Aug 12;13:1920486. doi: 10.3389/fnut.2026.1920486 (PMC13506818; doi:10.3389/fnut.2026.1920486)
Supplement: Supplementary file 1 [file Supplementary_File_1.docx]

Supplementary Material

# 1 Supplementary Tables

**SUPLEMMENTARY TABLES**

***Table S1***. Chickpea, teff and corn flour, xanthan gum, egg and water composition expressed in g/kg for each of formulation.

| **Formulation** | **Chickpea flour** | **Teff flour** | **Corn flour** | **Xanthan gum** | **Egg** | **Water** |
| --- | --- | --- | --- | --- | --- | --- |
| **1** | 338 | 0.0 | 338 | 0.0 | 169 | 155 |
| **2** | 0.0 | 338 | 338 | 0.0 | 169 | 155 |
| **3** | 338 | 338 | 0.0 | 0.0 | 169 | 155 |
| **4** | 338 | 0.0 | 338 | 14 | 0.0 | 311 |
| **5** | 0.0 | 338 | 338 | 14 | 0.0 | 311 |
| **6** | 338 | 338 | 0.0 | 14 | 0.0 | 311 |

***Table S2.*** *Proportion of different ingredients in each gluten-free fresh pasta formulation used in the study (g/kg).*

| **Formulation** | **F0** | **F10** | **F20** | **F25** | **F30** |
| --- | --- | --- | --- | --- | --- |
| **Other Flours: Carob Flour** | **100:0** | **90:10** | **80:20** | **75:25** | **70:30** |
| Chickpea flour | 338 | 304 | 270 | 253 | 236 |
| Teff flour | 338 | 304 | 270 | 253 | 236 |
| Carob flour | 0.0 | 68 | 135 | 169 | 203 |
| Xanthan gum | 14 | 14 | 14 | 14 | 14 |
| Water | 311 | 311 | 311 | 311 | 311 |

*Tagliatelle pasta was formulated with different blends of chickpea, teff, and carob flours: F0 (50:50:0); F10 (45:45:10); F20 (40:40:20); F25 (37.5:37.5:25); F30 (35:35:30).*

***Table S3.*** *Descriptive statistics of participants’ characteristics*

|  |  | **Non-Celiacs**  **(N=70)** | **Celiacs**  **(N=20)** |
| --- | --- | --- | --- |
| **Gender** | Female | 60 % | 50 % |
|  | Male | 40 % | 50 % |
| **Age** | 18-30 | 63 % | 45 % |
|  | 31-45 | 22 % | 15 % |
|  | 46-69 | 15 % | 40 % |
| **Frequency of pasta consumption** | Every day | 3 % | 0 % |
|  | At least once a week | 56 % | 55 % |
|  | At least once a month | 28 % | 35 % |
|  | Less than one a month | 13 % | 10 % |
| **Knowledge of carob products** | Yes | 22 % | 45 % |
|  | No | 78 % | 55 % |

**SUPLEMMENTARY FIGURES**

***Figure S1.*** Percentage of non-celiac participants who perceive the intensity of the attributes color, odor, texture and flavor of different plain pasta samples, as being “too little,” just-about-right (“JAR”), and “too much”. Evaluations were conducted for: A) plain pasta by non-celiacs, B) plain pasta by celiacs, and C) pasta with tomato sauce by celiacs. Tagliatelle pasta was formulated with different blends of chickpea, teff, and carob flours: **F0** (50:50:0); **F10** (45:45:10); **F20** (40:40:20); **F25** (37.5:37.5:25); **F30** (35:35:30).
